# Supplementary material for: Exploiting TLK1 and Cisplatin Synergy for Synthetic Lethality in Androgen-Insensitive Prostate Cancer
Source: Biomedicines. 2023 Nov 7;11(11):2987. doi: 10.3390/biomedicines11112987 (PMC10669050; doi:10.3390/biomedicines11112987)

Figure S1

Midpoint treatment group with 3mg/kg CPT+J54

CONTROL

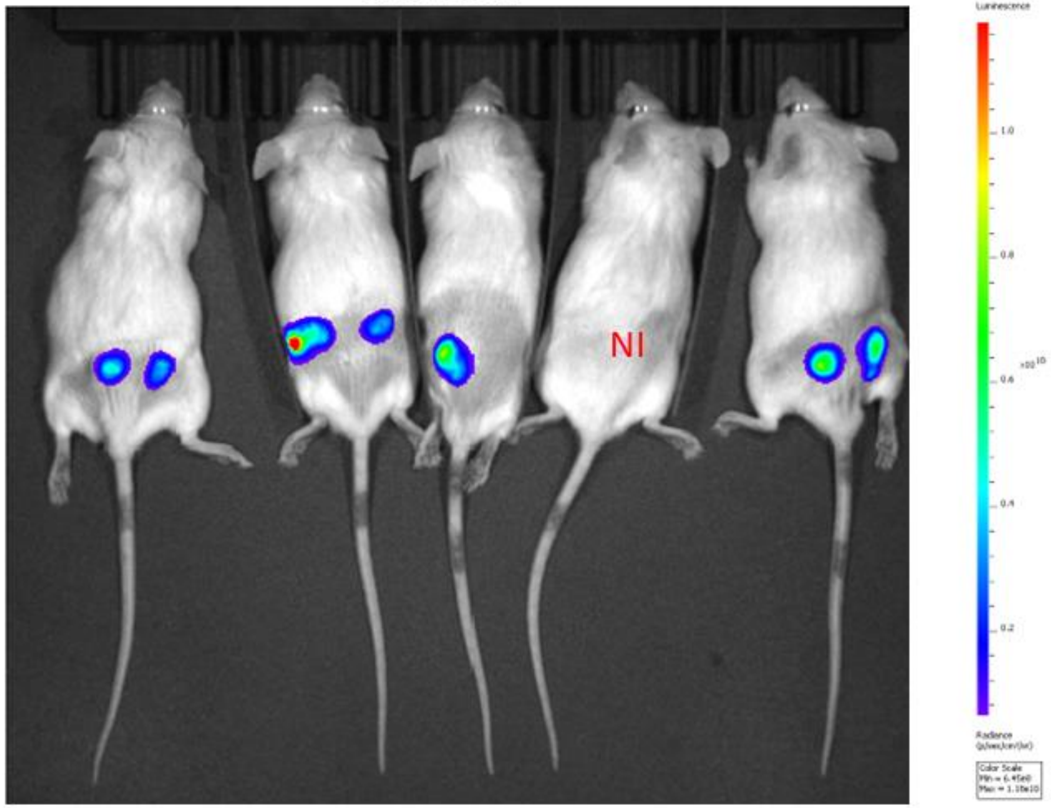

COMBO

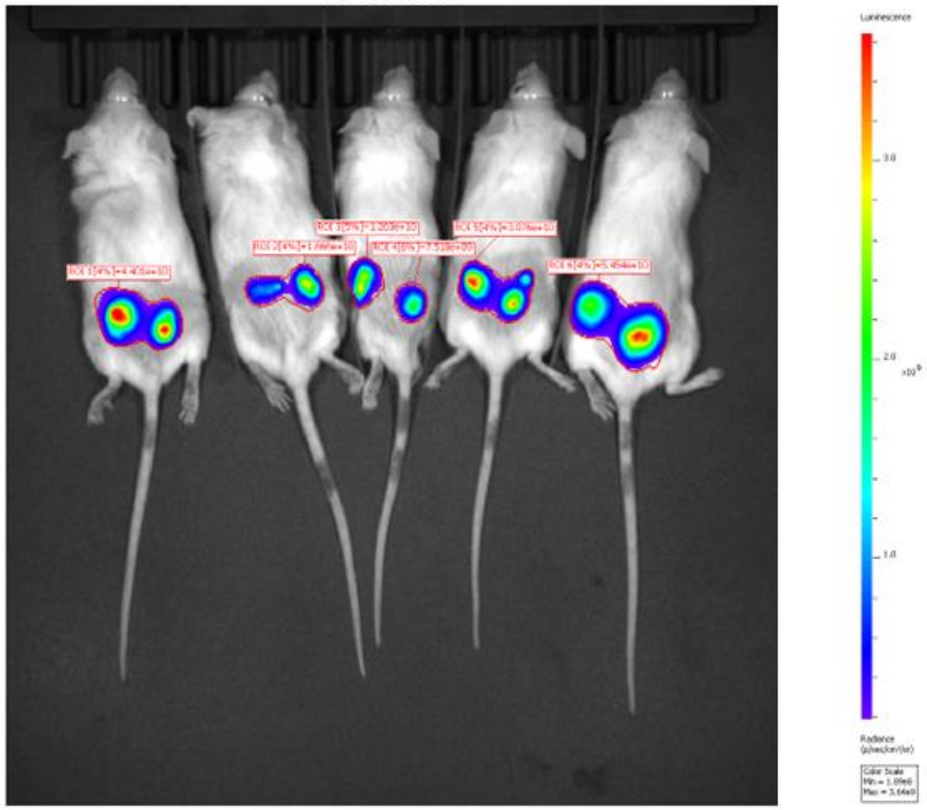

Figure S2. Phospho-NEK1 status and Cl-CAS3 in tumors from the treatment groups

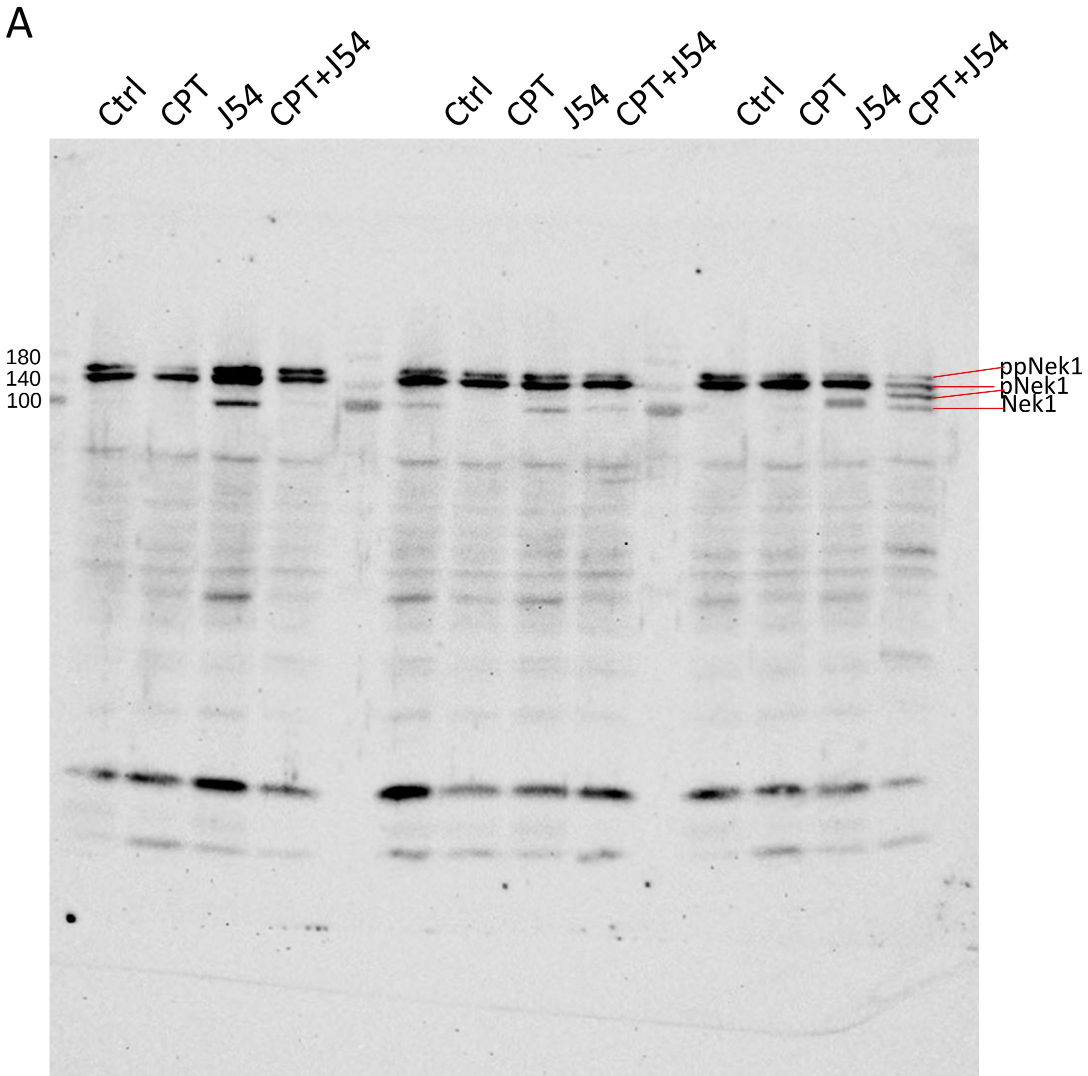

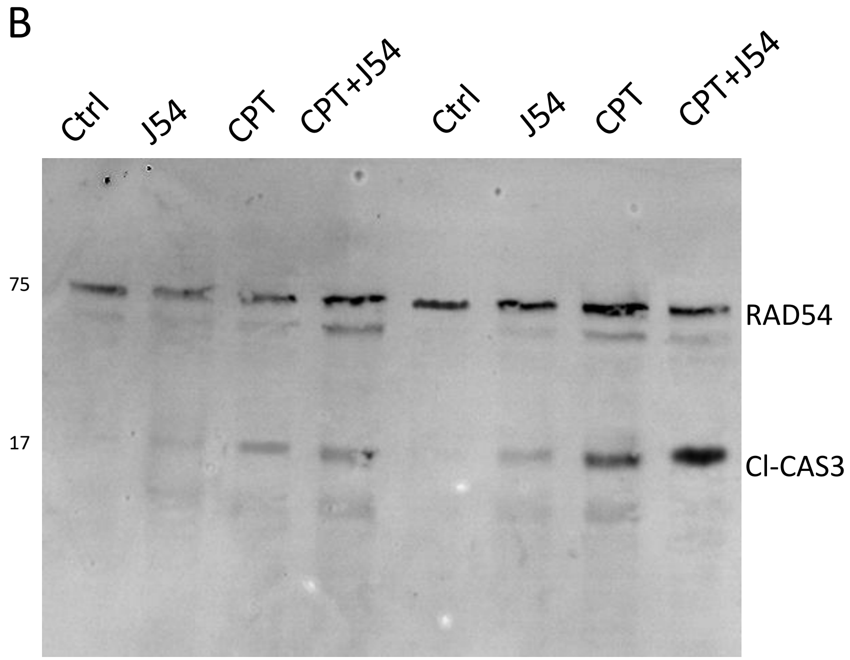

Figure S3.
Phospho-RAD54 status in tumors from the treatment groups

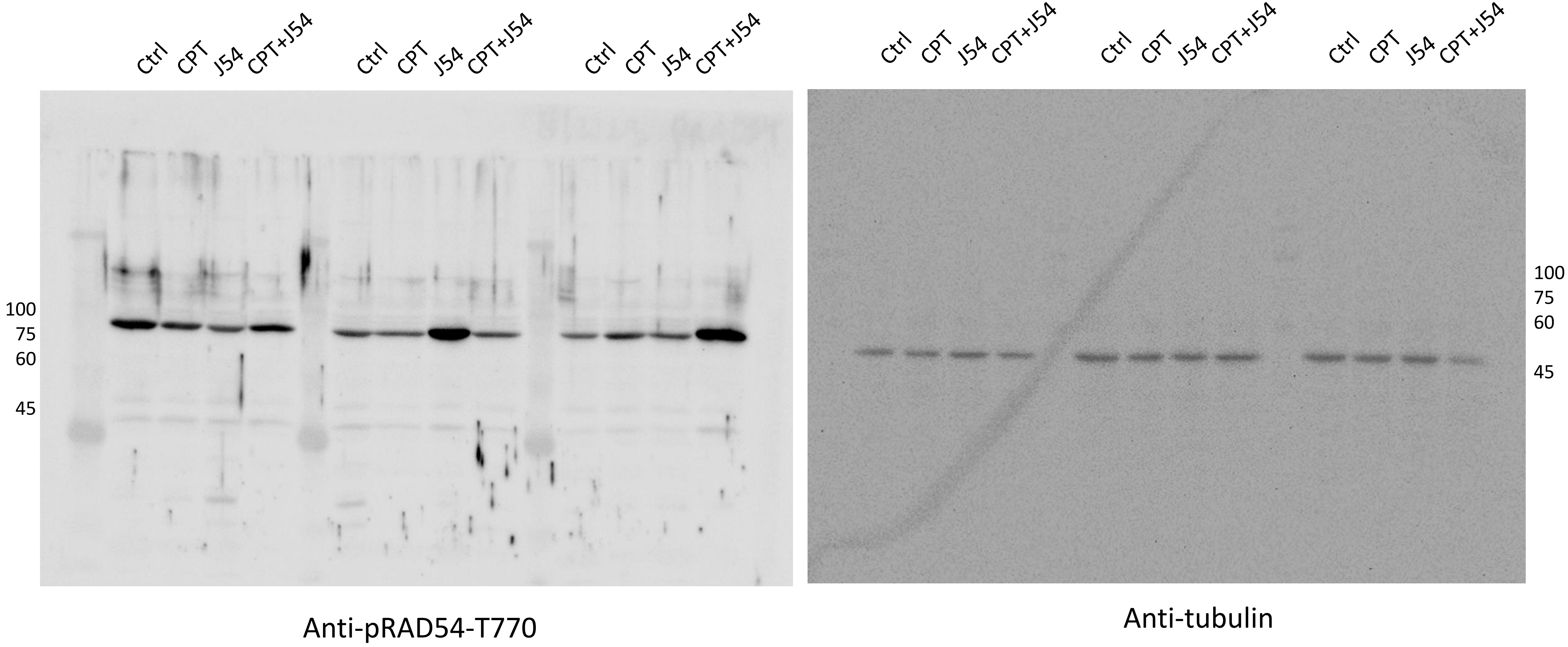

Figure S4

Fig.2 full blots

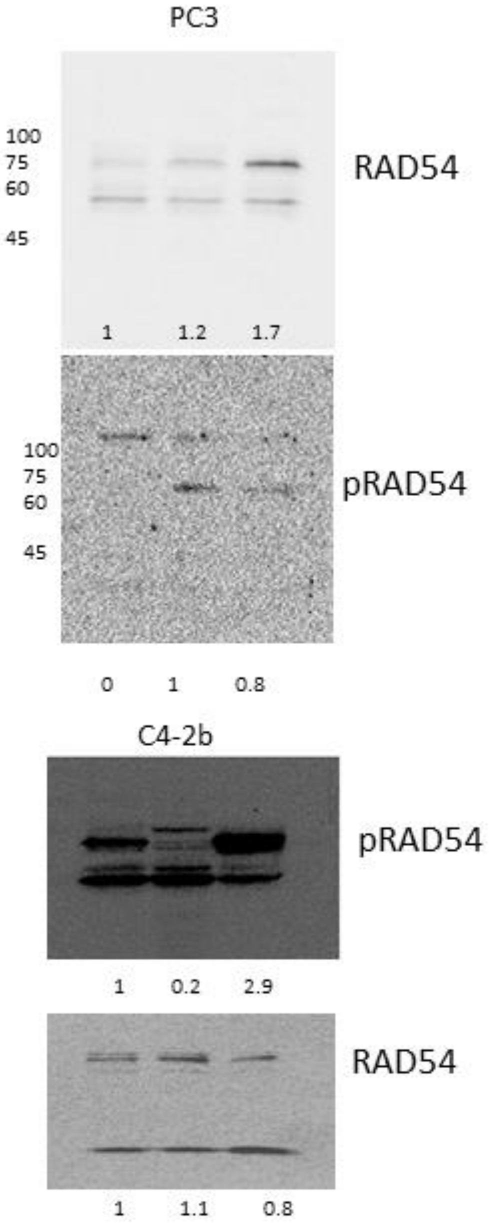

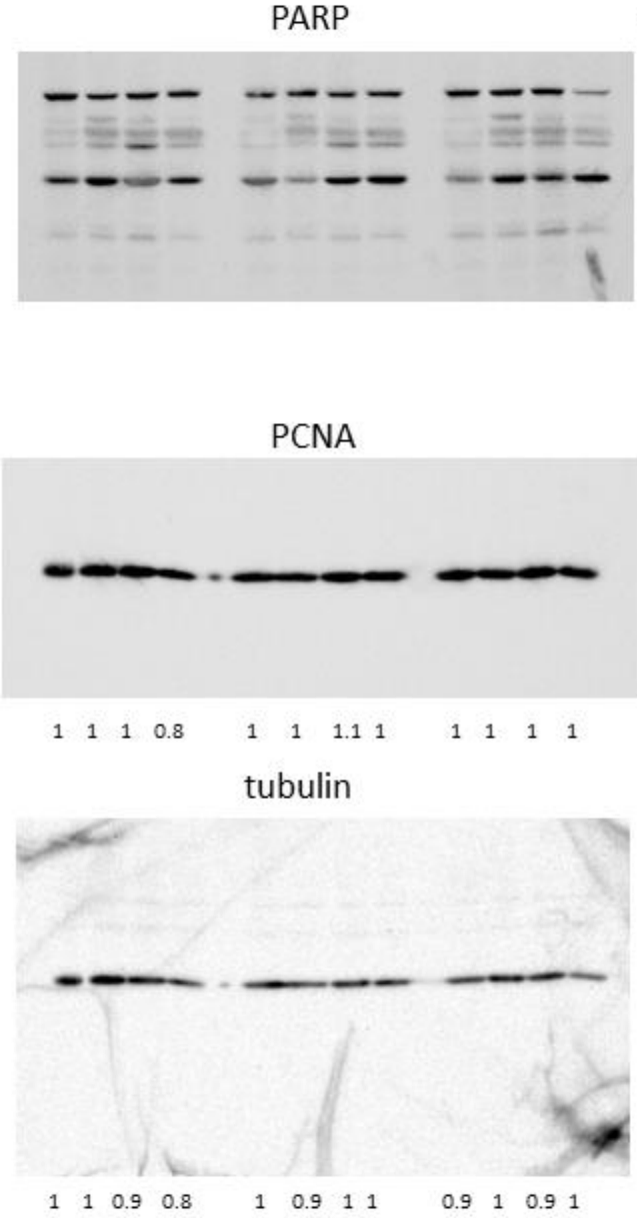

1	2	3	4	band
1	1	1	0.4	FL-PARP
0.2	1	0.3	0.3	Cl-P1
0.1	1	1.2	1	Cl-P2
0.1	1	1	1	Cl-P3

Fig.4 full blots

SI-FIG.3

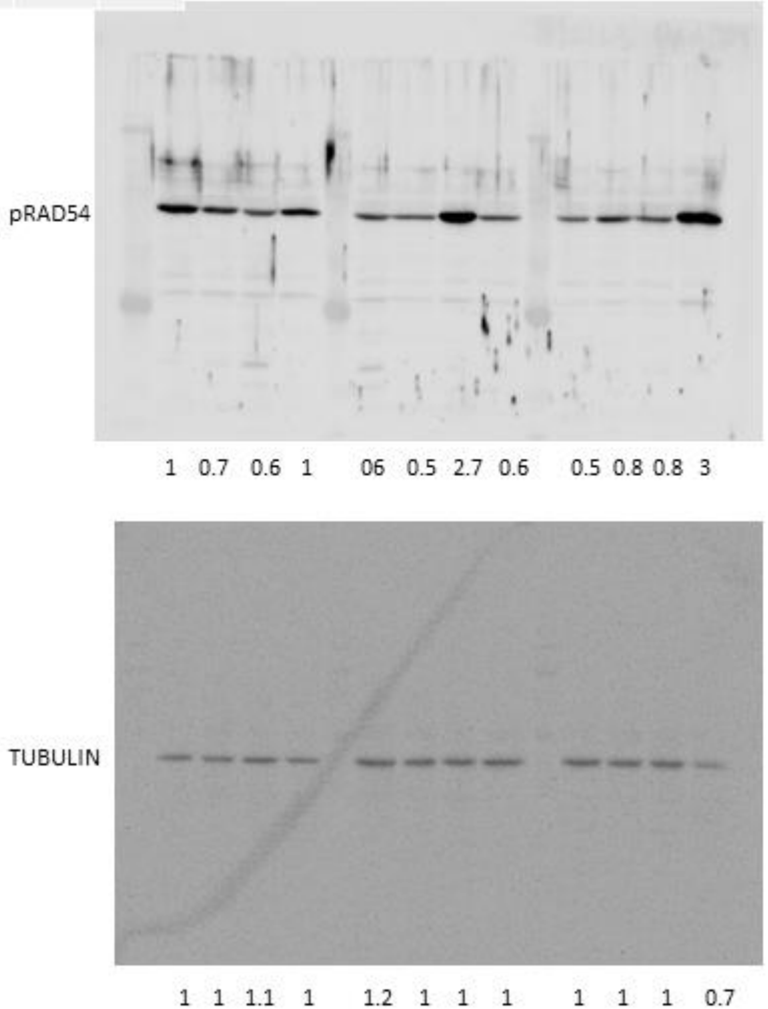

Figure S5

Selected Statistical SI data

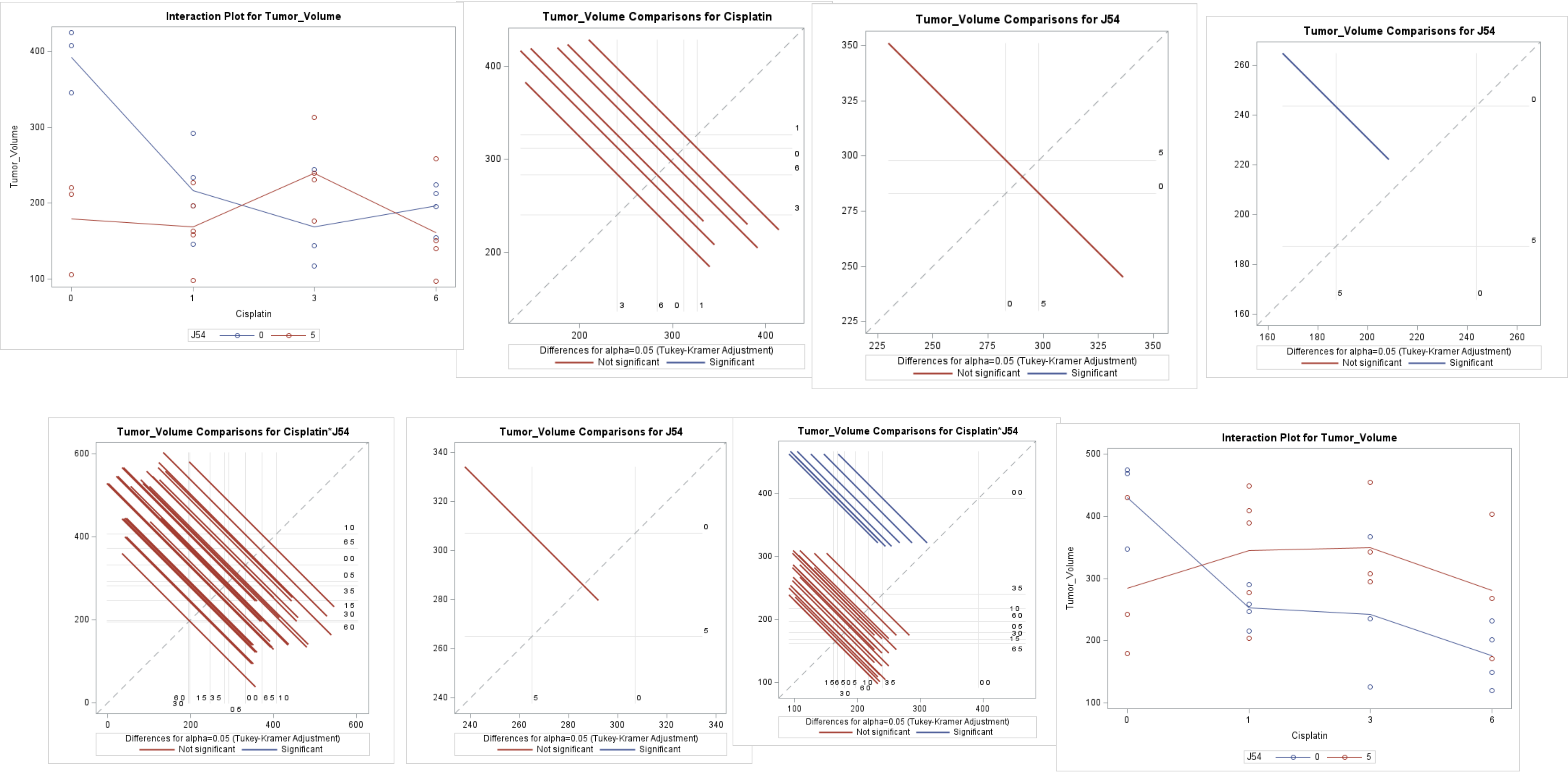

Supplement: Supplementary file 1 [file biomedicines-11-02987-s001.zip › biomedicines-2649895-supplementary.pdf]
